# TTGS: A topology-preserving method for vector reconstruction of plant cell wall networks

**DOI:** 10.1016/j.plaphe.2026.100294

**Published:** 2026-10-12

**Authors:** Zaibin Wang, Qingting Liu, Tao Wu, Jiaoli Jiang, Yan Zhang, Xueting Han, Xiaojuan Lin, Qing Zhou, Zhenghe Luo

**Affiliations:** a College of Engineering, South China Agricultural University, Guangzhou, China; b Guangdong Province Agricultural Technology Extension Center, Guangzhou, China; c College of Information Engineering, Northwest A&F University, Yangling, China

**Keywords:** Cell wall network, Microscopy image analysis, Plant-tissue microstructural reconstruction, Skeleton reconstruction

## Abstract

Plant cell wall architecture influences tissue mechanics and transport, but converting microscopy-derived masks into finite-element geometries requires both accurate centerline localization and preservation of network topology. We developed a topology-preserving thinning-based graph skeletonization method (TTGS) that converts binary cell wall masks into vectorizable node-edge graphs through skeleton extraction, node-cluster identification, path tracing, and connectivity-preserving linearization. Seven reconstruction methods were evaluated using the same binary masks from 12 cross-sectional micrographs of sugarcane (*Saccharum officinarum L.*) parenchyma, vascular bundles, and roots. Across all images, TTGS achieved a structure completeness of 44.04 ± 12.98%, a mean geometric deviation of 2.93 ± 1.04 px, a valid skeleton ratio of 94.49 ± 3.71%, and a breakage rate of 0%. Its unmatched internal endpoint count was 5.00 ± 5.38, while its closed-contour F1 and degree-aware junction F1 reached 93.73 ± 5.56% and 92.75 ± 3.54%, respectively. Among the methods satisfying the predefined short-fragment criterion, TTGS achieved the highest structure completeness and valid skeleton ratio and the lowest geometric deviation. A representative TTGS vector network was imported into finite-element software, reconstructed as a three-dimensional cell-scale model, and successfully solved to obtain a continuous stress distribution. These results show that TTGS balances geometric fidelity with topology preservation and supports the workflow from microscopy-derived masks to vector reconstruction and finite-element analysis.

## 1 Introduction

Plant vascular bundles, parenchyma, and other tissues exhibit highly ordered yet intricate spatial organization at the microscopic scale [1], [2]. Their cell wall geometry and intercellular connectivity influence tissue stiffness, anisotropic mechanical responses, and water and solute transport, thereby contributing to plant adaptation to external loads, drought, salinity, and other environmental factors [3], [4]. At the cellular scale, geometric traits such as wall thickness and lumen diameter interact with cellulose microfibril orientation and matrix viscoelasticity to shape mechanical behavior [5], [6], [7]. In crops such as sugarcane (*Saccharum officinarum L.*), the spatial organization of parenchyma and vascular bundle cell walls is intimately linked to sucrose loading efficiency, axial water transport, and lodging resistance-traits of direct agronomic and physiological significance. Accurate reconstruction of the geometry and topology of these networks from histological images is therefore essential for quantitative analysis, but suitable reconstruction tools remain limited.

Over the past two decades, multiscale mechanical modeling has become an important avenue for quantitative investigation of plant structure-function relationships [8], [9]. At the cellular scale, Wu et al. [10] reconstructed the geometry of individual fruit cells from micrographs and built finite-element models to analyze cell wall stress distributions and instability under varying turgor pressure. Pieczywek et al. [11] performed parametric modeling of plant microstructures using confocal microscopy to relate tissue-scale elastic moduli to local cell wall properties. At the tissue scale, Sayad et al. [12] proposed a machine-learning-assisted, Voronoi-based approach to model plant cell wall skeletons by converting microscopy images into two-dimensional topological structures, thereby providing geometric foundations for mechanical analysis of plant tissues.

Within these frameworks, the step from microscopy images to geometry useable for mechanical modeling serves as a key bridge between experimental observation and multiscale modeling. Histological section images capture the spatial configurations of cells and tissues; transforming the cell wall network into identifiable geometric entities requires robust segmentation, topology-faithful skeleton extraction, and conversion to vector networks and meshed geometries [13], [14], [15]. Foreground-background separation can be achieved using global or adaptive thresholding, edge detection, and region growing. Otsu’s method [16], which maximizes between-class variance, is widely used for threshold selection, whereas adaptive histogram equalization and CLAHE improve contrast under non-uniform illumination [17]. Region-based reconstruction commonly uses Voronoi models derived from cell centroids to approximate spatial distributions and adjacency relations [18], [19]. Morphological thinning compresses wall regions into one-pixel-wide skeletons, with the Zhang-Suen algorithm representing a classical pixel-level approach [20]. Deep architectures such as U-Net [21] and the nested multiscale U2-Net [22] can further improve pixel-level localization. However, high pixel-level agreement does not necessarily guarantee a continuous network that can be directly vectorized for mechanical modeling.

Despite their success under specific imaging conditions and for certain specimens, these methods share limitations when applied to multiscale mechanical modeling. Reconstruction quality often depends heavily on preprocessing: uneven illumination and staining cause strong regional variations in gray-level distributions, making global or local thresholds difficult to standardize and requiring manual parameter tuning, which compromises consistency and reproducibility across samples [23], [24]. Segmentation results may retain noise structures inside lumens or in the background, which can be converted during skeletonization into non-physical branches or redundant connections, undermining the topological plausibility of skeleton networks [25]. Moreover, many existing methods impose constraints on cell morphology and spatial uniformity; when cells are irregular, wall thickness varies markedly, or local structures are distorted, reconstruction performance degrades in both geometric consistency and topological correctness, limiting generality and portability [14]. These technical limitations directly impede biological inquiry that depends on accurate wall network reconstruction: how does cell wall topology vary across tissue types with distinct physiological functions, and how do wall geometry parameters shift in response to developmental or environmental cues? Therefore, it is necessary to develop a microscopy image reconstruction method that is motivated by both multiscale modeling needs and biological inquiry, more adaptive to varying imaging conditions, and capable of stably preserving cell wall topology for downstream physiological and biomechanical analysis.

## 2 Materials and methods

### 2.1 Experimental materials and microscopy image acquisition

Microscopic images were acquired from cross-sectional histological sections of sugarcane stems and roots. Five mature plants of the sugarcane cultivar Guitang 49 were selected from the Subtropical Crops Research Institute in Zhanjiang, Guangdong, China. The selected plants had an intact appearance, straight stalks, and no visible symptoms of pests or diseases. Rectangular tissue blocks (2 mm × 2 mm × 10 mm) were excised from different parts of the samples and fixed in formalin-acetic acid-alcohol fixative to preserve cell morphology and tissue connectivity. Samples were then processed following standard paraffin sectioning procedures, including dehydration, clearing, paraffin infiltration, embedding, sectioning, and mounting. Staining was applied to enhance contrast between cell walls and lumens, producing stable grayscale differences across the RGB channels for subsequent segmentation and quantitative structural analysis.

The prepared sections were observed using a Motic BA210 optical microscope, and high-resolution micrographs were acquired using the integrated camera system and Image-Pro Plus software. For each of the three representative tissue regions—parenchyma, vascular bundles, and roots—four non-overlapping fields of view were randomly selected from sections obtained across the five plants, resulting in a total of 12 micrographs for subsequent skeleton reconstruction. These fields of view were treated as image-level samples for method evaluation rather than as independent biological replicates.

### 2.2 Microscopy image structural reconstruction algorithm

#### 2.2.1 Overall framework of the proposed topology-preserving thinning-based graph skeletonization method

We developed a topology-preserving thinning-based graph skeletonization (TTGS) framework for tissue geometry reconstruction, aiming to systematically parse the topological network of cell walls from micrographs and convert it into a computational physical model. As illustrated in Fig. 1, the pipeline consists of four stages: image normalization and enhancement, adaptive threshold segmentation and morphological denoising, skeleton extraction and node identification, and path tracing with graph construction and export.

**Fig. 1:**
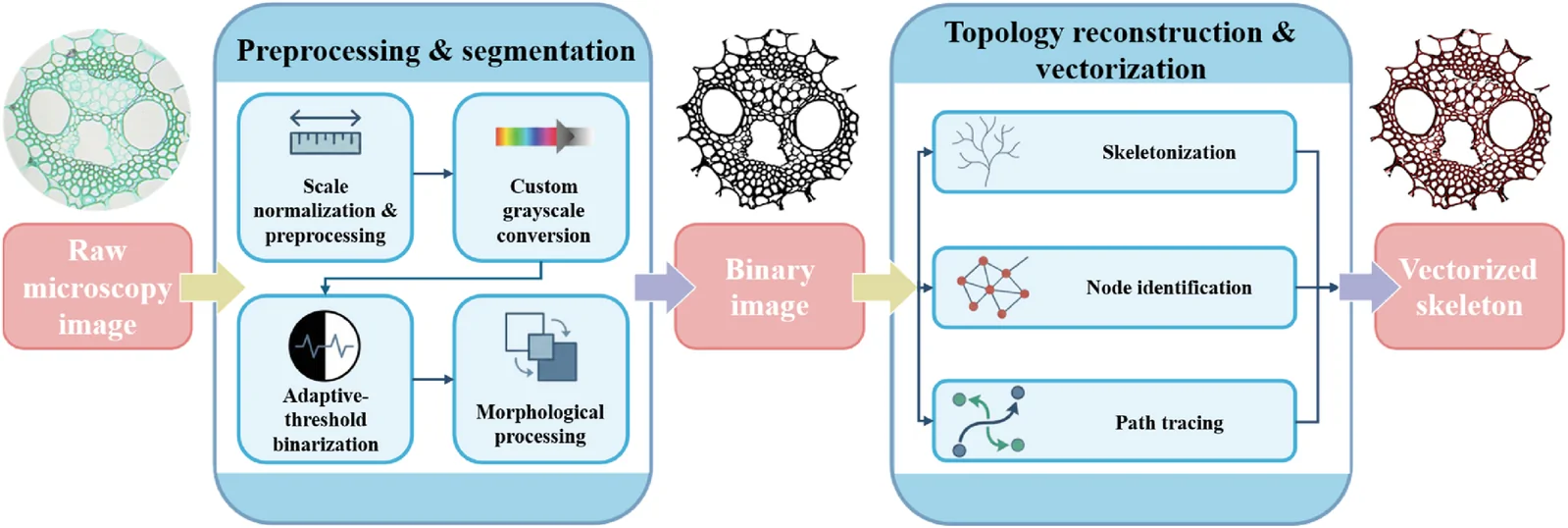
Overall image-processing workflow.

#### 2.2.2 Preprocessing and segmentation

To obtain a cell wall binary result with good connectivity and reduced noise, preprocessing and binarization were performed in the following order: scale normalization and enhancement, optimization of grayscale representation, adaptive segmentation, and morphological cleaning.

First, the original RGB micrograph I_0_ ∈ ℝ^H^×^w^×^3^ was converted to a grayscale image Igray using the standard grayscale conversion implemented in MATLAB. The grayscale image was then normalized to [0, 1]. To reduce local contrast fluctuations caused by non-uniform illumination, contrast-limited adaptive histogram equalization (CLAHE) was applied to I_gray_ to produce an enhanced grayscale image I_enh_. This step increased the grayscale separation between cell walls and lumens in darker regions, thereby stabilizing subsequent thresholding.

Binarization adopted blockwise Otsu thresholding to accommodate intensity gradients within a single image. The enhanced image I_enh_ was divided into an N_b_ × N_b_ grid of non-overlapping blocks, where N_b_ controls the spatial scale of threshold estimation. For each non-constant block Ω_b_, the threshold was calculated independently using Otsu’s method:

(1) $$T_{b}=\text{Otsu}\left(I_{\text{enh}}\;|\;\Omega_{b}\right)$$

where T_b_ is the threshold calculated for block Ω_b._ For a constant-intensity block, the Otsu threshold calculated from the complete enhanced image was used as a fallback. Under the prior that the cell wall appears darker, the initial binary image was defined as:

(2) $$B_{0}\left(x,y\right)=\left\{ \begin{array}{cc} 0, & I_{\text{enh}}\left(x,y\right)\leq T_{b}\\ 1, & I_{\text{enh}}\left(x,y\right)\;>\;T_{b} \end{array}\right.$$

where 0 represents cell walls and 1 represents cell lumens or background. The binary results from all blocks were assembled to produce the complete binary image.

At the denoising stage, connected-component area filtering was performed. The binary image was inverted so that black regions were treated as foreground components. Components with areas below the adjustable threshold Amin were removed, and the image was then inverted again to produce the final cell wall binary image B_final_. The same B_final_ images were used as the common inputs for all seven reconstruction methods.

#### 2.2.3 Topology reconstruction and vectorization

For topology-preserving vector network reconstruction, the binary cell wall region was first skeletonized to obtain a one-pixel-wide skeleton. Because skeletonization can introduce small spurs and short spurious branches that directly affect node localization and connectivity inference, skeleton connected components were analyzed by length and branches shorter than the threshold L_min_ were removed, yielding a more stable simplified skeleton S′(x, y).

During node identification, skeleton pixels were treated as vertices of an undirected graph. For a given pixel (x, y), the number of skeleton neighbors in its 8-neighborhood was counted:

(3) $$d\left(x,y\right)=\sum_{\left(u,v\right)\in N_{8}\left(x,y\right)}S^{\prime }\left(u,v\right)$$

where N_8_(x, y) denotes the 8-neighborhood. Pixels were classified by degree: d(x, y) = 2 indicates an internal path pixel, whereas d(x, y) ≠ 2 indicates a node pixel (endpoint d = 1 or junction d ≥ 3). Notably, nodes detected by this criterion often appear as a small connected region rather than a single pixel. Therefore, all node pixels were grouped into connected components C_1_, …, C_K_, and the geometric centroid of each node cluster was taken as the initial node location, averaging out the influence of local pixel noise. Furthermore, if the centroid distance between two node clusters was below the threshold d_min_, they were likely fragments of the same physical node caused by skeletonization or spur removal; adjacent nodes were thus merged to obtain a stable node set V = {v_1_, …, v_N_}, as shown in Fig. 2(a). This “degree-based detection-node clustering-centroid localization-nearest-neighbor merging” procedure enables node localization to reflect true topological changes while remaining robust to local zigzags and pixel discretization.

**Fig. 2:**
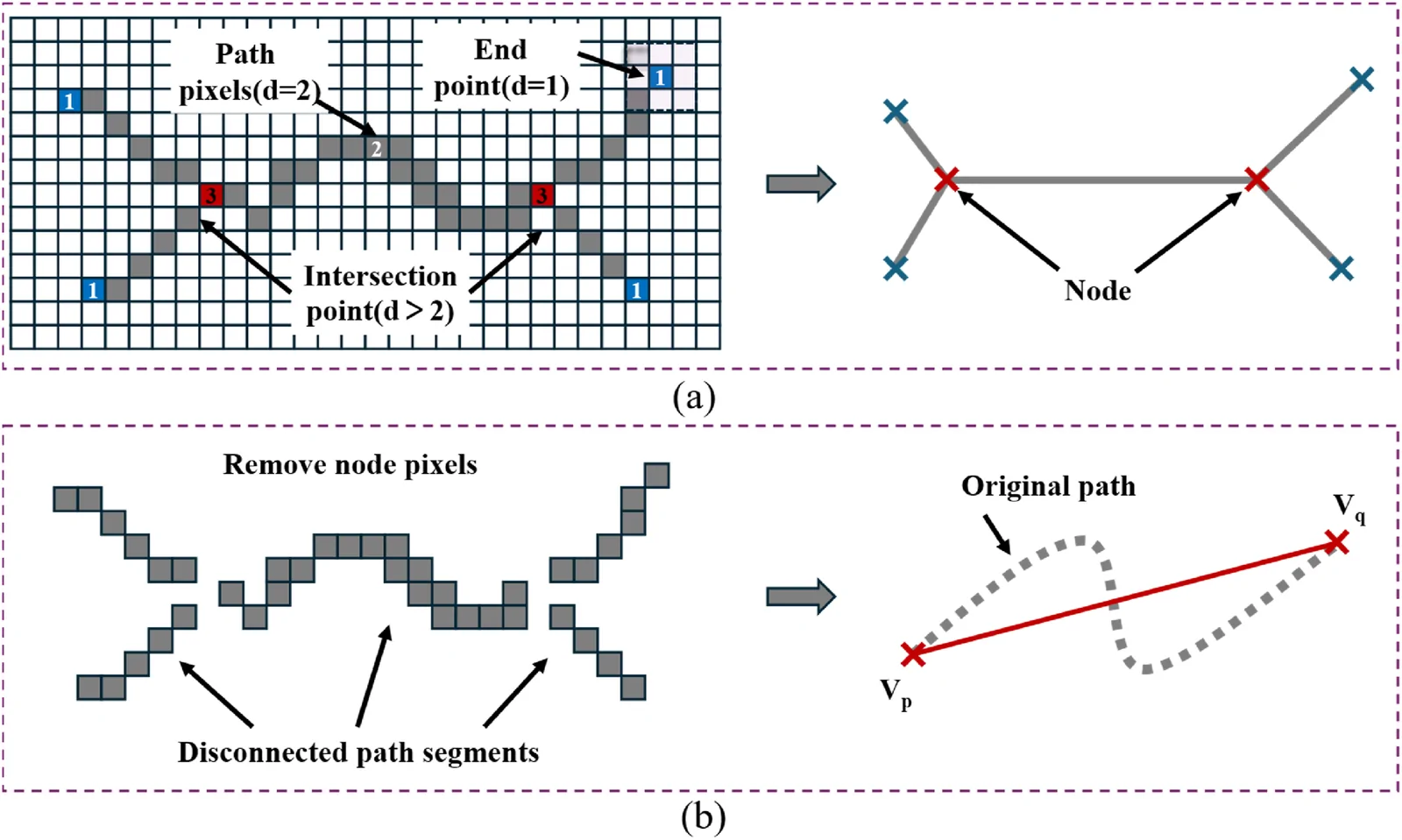
Node identification and linearization: (a) node identification process, (b) linearization process.

During linearization, the goal is to convert pixel-level paths into vectorizable geometric elements without altering topological connectivity. Specifically, node-cluster pixels were removed from the skeleton, retaining only path pixels, so that the skeleton decomposes into a set of pairwise disjoint connected paths Γ_j_. For each path, its two endpoints were determined by its contact relationships with the node set V, yielding the corresponding nodes v_p_ and v_q_, thereby ensuring that edge endpoints are consistent with the node set. A least-squares line fit was then performed, and a line segment was used to connect v_p_ and v_q_, as shown in Fig. 2(b). Given the computational demands of subsequent tissue-scale finite-element simulations, this straight-line representation was adopted to limit geometric complexity, mesh density, and computational cost. Although this simplification may underrepresent local curvature-related mechanical behavior in highly curved cell walls, polyline or spline fitting with a limited number of control points can be introduced in future implementations to preserve local curvature while controlling model complexity.

Finally, based on the obtained graph structure G = (V, E), the network was exported to vector formats such as DXF and SVG. Each edge was stored by its start-end node coordinates and, when needed, a sequence of control points, enabling direct use as a geometric skeleton input for subsequent modeling while preserving the same topological connectivity as the pixel-level skeleton.

### 2.3 Baseline methods and evaluation metrics

#### 2.3.1 Baseline methods

To compare skeleton-generation mechanisms under identical input conditions, seven methods were grouped into four categories: (i) CVT and its derivatives, including Centroidal Voronoi Tessellation (CVT), Finite-edge Centroidal Voronoi Tessellation (FECVT), and Optimized Distance Weighted Tessellation (ODWT); (ii) the classical Zhang-Suen thinning algorithm; (iii) the deep-learning-based U-Net and U2-Net models; and (iv) the proposed TTGS method. All methods received the same binary cell wall masks and produced skeleton representations for evaluation (Table 1). The default parameter settings, parameter functions, and applicable adjustment ranges for all seven methods are summarized in Supplementary Table S1. The source code and detailed implementation and parameter settings for all evaluated methods have also been deposited in the accompanying data repository to facilitate reproducibility.

**Table 1:** Skeleton network construction methods.

Method name	Abbreviation	Principle overview
Centroidal Voronoi Tessellation [19]	CVT	The centroid of each connected lumen region is used as a generator point. Delaunay triangulation is performed, and the circumcenters of triangles are taken as Voronoi vertices; adjacent circumcenters are connected to form the cell wall network.
Finite-edge Centroidal Voronoi Tessellation [26], [27]	FECVT	Based on CVT, boundary finite-ization is applied. Only interior Voronoi edges are retained, and unbounded rays generated at the convex-hull boundary are trimmed/filtered, so that the output network is closed within the image domain and is amenable to engineering vectorization.
Optimized Distance Weighted Tessellation [28]	ODWT	Using Delaunay triangles as basic units, a distance-weighting factor related to cell area is introduced to replace the standard Voronoi circumcenter vertex with a weighted bisector point; adjacent weighted points of neighboring triangles are connected to form the cell wall network.
Zhang-Suen thinning [20]	Zhang-Suen	Iterative deletion rules thin the binary wall mask to a one-pixel-wide skeleton while preserving local pixel connectivity; the method does not explicitly repair disconnected components or enforce closed network geometry.
U-Net [21]	U-Net	An encoder-decoder architecture with skip connections predicts fine skeleton details from the common binary mask; pixel-level prediction does not explicitly guarantee closed or non-fragmented output networks.
U2-Net [22]	U2-Net	A nested U-structure with residual U-blocks captures multiscale contextual information and predicts skeleton pixels from the common binary mask; global network connectivity is not imposed as a hard constraint.
Topology-preserving thinning-based graph skeletonization	TTGS	A one-pixel wall skeleton is converted into an explicit node-edge graph through node-cluster identification, path decomposition, and tracing; connectivity-preserving linearization enables engineering-oriented vector export.

#### 2.3.2 Evaluation metrics

To evaluate skeleton quality under a common reference, an initial one-pixel skeleton was automatically extracted from each binary cell wall mask and then manually inspected and corrected to remove spurious branches, repair evident discontinuities, and resolve connections inconsistent with the visible wall network. The resulting reference skeletons were subsequently independently verified in a blinded manner by a second researcher experienced in plant tissue image analysis. All 12 reference skeletons were reviewed. Seven were accepted without modification, whereas five required localized corrections. Image-level verification outcomes, correction categories, and corresponding sample identifiers are provided in Supplementary Table S2. The verified skeleton was used as the reference skeleton S_ref_ for every method. The reconstructed skeleton produced by each method was denoted S_pred_, and the shortest Euclidean distance from a point to a set was defined as follows:

(4) $$d\left(x,S\right)=\min_{s\in S}{\left\|x\;-\;s\right\|}_{2}$$

Seven metrics were used to characterize skeleton reconstruction performance, as summarized in Table 2. Structure completeness (SC), mean geometric deviation (GD_Mean), and valid skeleton ratio (SER_Acc) were used to evaluate structural coverage and geometric agreement. Unmatched internal endpoints (UIE), closed-contour F1 (CC_F1), and degree-aware junction F1 (DAJ_F1) were used to characterize internal discontinuities, closed-contour preservation, and junction-level topological consistency, respectively. Breakage rate (BR) quantifies the occurrence of short fragmented components and was used as a practical screening criterion. BR = 0 indicates that no additional short-fragment correction is required, whereas BR > 0 indicates that further connectivity correction is needed. Together, these metrics provide complementary assessments of geometric fidelity, fragmentation, and network topology.

**Table 2:** Evaluation metrics.

Metric	Formula	Meaning
Structure completeness (SC)	$SC=\frac{\left|\left\{x\in {\;S}_{\text{ref}}|d\left(x,{\;S}_{\text{pred}}\right)\leq \varepsilon \right\}\right|}{\left|S_{\text{ref}}\right|}$	Proportion of pixels in the reference skeleton whose distance to the reconstructed skeleton ≤ ε (px) satisfies the tolerance, measuring coverage completeness (missed detections). SC ∈ [0, 1]; higher is better.
Geometric deviation (GD_Mean)	$\text{GD}\_\text{Mean}=\frac{1}{\left|S_{\text{pred}}\right|}\sum_{y\in S_{\text{pred}}}d\left(y,{\;S}_{\text{ref}}\right)$	Mean shortest distance (px) from pixels of the reconstructed skeleton to the reference skeleton, measuring geometric positional deviation. GD_Mean ≥ 0; lower is better.
Breakage rate (BR)	$\text{BR}=\frac{\left|\left\{i|l_{i}\;<\;L_{b}\right\}\right|}{\max\left(1,N\right)}$	Proportion of 8-connected skeleton components whose lengths are smaller than L_b_ (12 px), measuring the occurrence of short fragmented components. BR ∈ [0, 1]; lower is better.
Valid skeleton ratio (SER_Acc)	$\text{SER}\_\text{Acc}=\frac{\left|S_{\text{pred}}\cap B_{\text{final}}\right|}{\left|S_{\text{pred}}\right|}$	Proportion of reconstructed skeleton pixels that fall within the B_final_ mask, measuring the plausibility of the support region (fewer spurious edges). SER_Acc ∈ [0, 1]; higher is better.
Unmatched internal endpoints (UIE)	$U_{E}=|E_{\text{pred}}^{\mathrm{int}}|\;-\;M_{E}$	Number of predicted 8-neighbor endpoints located more than 3 px from both the image boundary and the reference-domain boundary that cannot be matched one-to-one to a reference endpoint within 3 px. Lower is better.
Closed-contour F1 (CC_F1)	$P_{c}=\frac{M_{c}}{N_{\text{pred}}^{c}}$ $R_{c}=\frac{M_{c}}{N_{\text{ref}}^{c}}$ $F_{1,c}=\frac{2P_{c}R_{c}}{P_{c}+R_{c}}$	Closed faces are extracted from the 4-connected complement of each 8-connected skeleton after excluding boundary-touching background. Predicted and reference faces are matched one-to-one at IoU ≥ 0.5. Higher is better.
Degree-aware junction F1 (DAJ_F1)	$P_{j}=\frac{M_{j}}{N_{\text{pred}}^{j}}$ $R_{j}=\frac{M_{j}}{N_{\text{ref}}^{j}}$ $F_{1,j}=\frac{2P_{j}R_{j}}{P_{j}+R_{j}}$	Predicted and reference junction clusters are matched one-to-one within 3 px only when their graph degrees agree. Higher is better.

## 3 Results and analysis

### 3.1 Binarization results and analysis of input geometric features

To ensure comparability and reproducibility across reconstruction strategies, all seven methods used the same cell wall binary mask B_final_ obtained from the common preprocessing pipeline. Fig. 3(a)-(c) shows the original micrographs of three representative tissue regions, and Fig. 3(d)-(f) shows the corresponding binary masks, where black regions represent the cell wall network and white regions represent lumens and background.

**Fig. 3:**
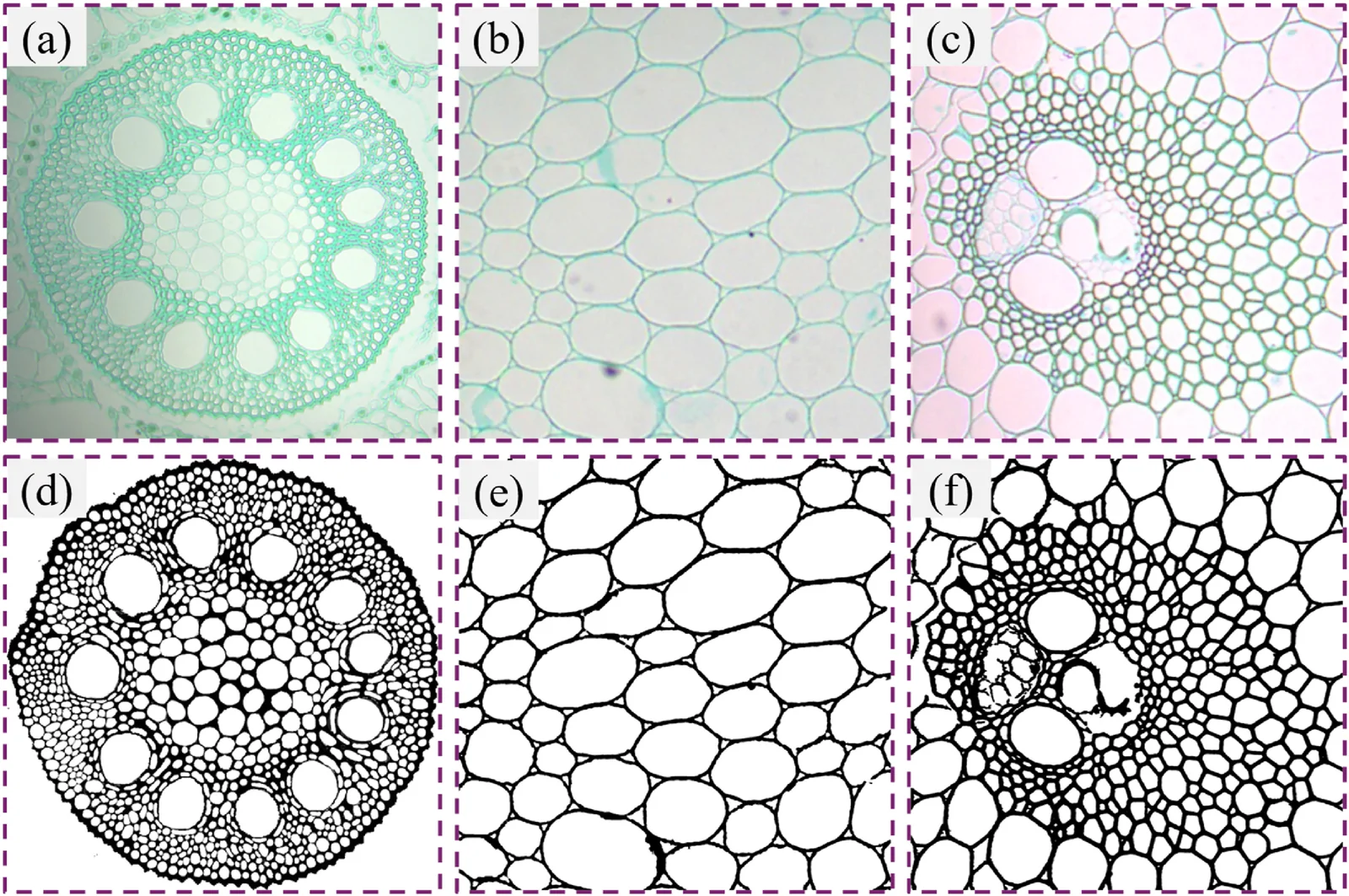
Binarization results for three representative tissue regions: (a)–(c) original microscopy images, (d)–(f) corresponding binarization results (black: cell wall network; white: cell lumens and background).

The binarization results for the three tissue types all exhibit a clear separation between “cell wall” and “cell lumen”, providing a unified and comparable input for subsequent skeleton extraction and network reconstruction. However, the tissue regions differ markedly in geometric scale, connectivity, and noise interference, leading to different reconstruction challenges.

The root cross-sections show the most typical hierarchical and multiscale characteristics: dense networks of small cell walls on the periphery, with multiple much larger cavities inside. The wall network exhibits a pronounced “dense-sparse” spatial gradient, and features numerous junctions, short edge segments, and frequent multi-branch structures, representing a complex topological scenario with high connectivity and high node density. In contrast, parenchyma regions mainly consist of more regular cell shapes, larger lumens, smoother boundaries, and more uniform overall arrangements, resulting in geometries closer to regular polygonal networks. However, their cell walls are thin and locally non-uniform in thickness, which may appear in binary images as fine wall filaments with mild zigzags or local breaks, making them more sensitive to connectivity preservation and spur suppression. Vascular-bundle regions superimpose a dual-scale structure of “high-density clusters of small cells” and “interspersed large cavities”, with denser junctions and more prominent node clusters. In addition, occasional irregular dark blobs or damaged residues can be observed; such artifacts more readily induce spurious branches or cross-lumen shortcut edges, imposing higher requirements on spurious-edge suppression and topological stability [9], [29].

From a biological perspective, these geometric differences are consistent with the distinct structural and physiological functions of the three tissue types. The densely connected small-cell network in roots may contribute to tissue integrity and provide structural pathways for radial water and solute transport. The larger lumens and more regular organization of parenchyma are consistent with storage and tissue-deformation functions. By contrast, the dual-scale architecture of vascular bundles reflects the coexistence of axial transport conduits and mechanically reinforcing surrounding tissues. Although physiological functions were not measured directly, preserving these tissue-specific architectures is essential for subsequent quantification of cell geometry, lumen distribution, and network connectivity.

### 3.2 Cell wall extraction performance and parameter sensitivity of TTGS

Fig. 4 presents the skeletonization results produced by TTGS for three representative tissues (vascular bundles, parenchyma, and root tissues). The following characteristics can be observed visually:

**Fig. 4:**
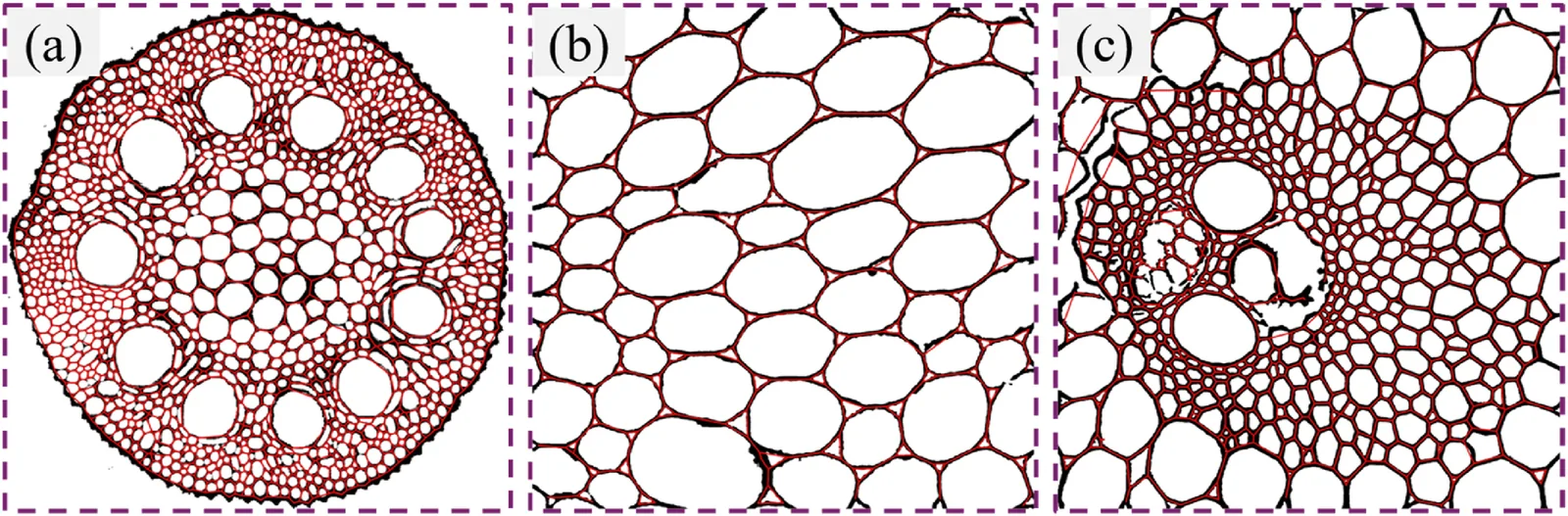
Skeletons generated by TTGS in three representative regions (red lines: TTGS skeletons; black regions: cell wall network): (a) root tissues, (b) parenchyma, (c) vascular bundles.

Overall, TTGS skeletons showed good spatial agreement with the cell wall masks, with most skeleton lines following the centers of wall regions and few long-distance shortcut segments crossing the lumens. The reconstructed networks generally maintained continuous cell boundaries with relatively few fragmented or isolated short segments, indicating good wall adherence and network continuity. Across tissue types, TTGS preserved fine branches in the multiscale root network (Fig. 4(a)), produced relatively smooth and complete skeletons in parenchyma (Fig. 4(b)), and maintained clear junction relationships in the more densely connected vascular-bundle regions (Fig. 4(c)). These observations indicate consistent qualitative performance across tissues with different structural complexities and support subsequent node-edge parameterization and vector export.

To further evaluate the robustness of TTGS to parameter selection, sensitivity analyses were conducted for L_min_ (0–60 px) and d_min_ (0–4 px) across all 12 images. As shown in Supplementary Fig. S1, increasing L_min_ from 0 to 30 px markedly reduced UIE from 100.75 to 5.00 and increased DAJ_F1 from 90.29% to 92.75%, while GD_Mean decreased from 3.29 to 2.93 px. Over the same range, SC, SER_Acc, CC_F1, and BR remained relatively stable, indicating that short-branch removal substantially reduced abnormal internal endpoints without appreciably altering the overall skeleton structure. Further increases in L_min_ produced only limited changes in most metrics. In comparison, d_min_ had a smaller overall influence (Supplementary Fig. S2), although DAJ_F1 decreased to 90.77% at d_min_ = 4 px, suggesting that excessive node merging may adversely affect junction-level topology. Overall, TTGS remained relatively stable over the tested parameter ranges, with L_min_ primarily affecting short-branch removal and dmin mainly influencing junction-level topology. Based on these results, L_min_ = 30 px and d_min_ = 1 px were retained for subsequent evaluation.

### 3.3 Method-specific reconstruction characteristics and qualitative comparison

The seven methods represent four distinct skeleton-reconstruction strategies: (i) CVT and its derivatives infer wall networks from lumen centroids or weighted generator points; (ii) Zhang-Suen directly thins the binary wall mask; (iii) U2-Net and U-Net predict wall centerlines using deep encoder-decoder architectures; and (iv) TTGS combines pixel-level thinning with explicit node identification, path decomposition, and graph reconstruction. Their different geometric assumptions and connectivity mechanisms produced distinct characteristics in wall localization, structural coverage, and network integrity.

CVT and its derivatives construct networks from lumen centroids or weighted generator points. CVT therefore produces a connected but strongly regularized partition whose edges can deviate from observed wall centerlines, particularly where large and small cells coexist (Fig. 5(a)). FECVT removes or truncates unbounded boundary rays and improves boundary usability (Fig. 5(b)), but it does not fundamentally correct internal position errors inherited from centroid-driven construction. ODWT introduces distance- or area-related weighting and therefore adapts better to unequal cell sizes (Fig. 5(c)), although its edge positions remain sensitive to lumen segmentation and local scale variation. These methods favour network connectivity, but connectivity alone does not guarantee anatomical fidelity.

**Fig. 5:**
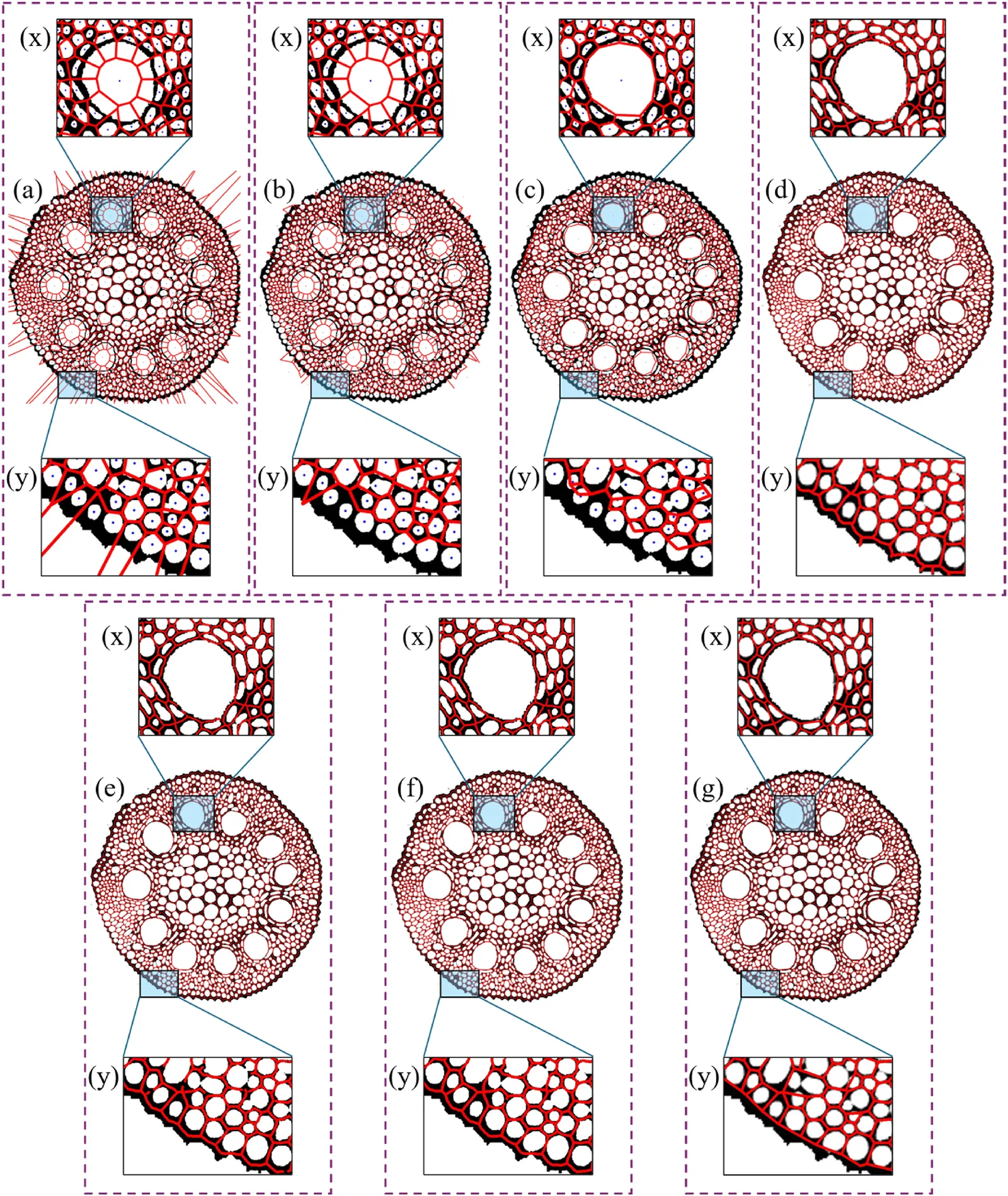
Examples of skeletons overlaid on the original image for different methods: (a) CVT, (b) FECVT, (c) ODWT, (d) Zhang-Suen, (e) U-Net, (f) U2-Net, and (g) TTGS. The middle panel shows the complete reconstruction result, and the upper and lower insets show magnified views around the vessel region (large lumen) and near the tissue boundary, respectively. Red lines indicate reconstructed skeletons or networks, black regions indicate the cell wall mask, blue dots indicate cell centroids used by the Voronoi-based methods, and light-blue boxes indicate the magnified regions.

The classical Zhang-Suen algorithm directly thins the binary wall mask and can therefore retain close pixel-level correspondence with the observed wall regions (Fig. 5(d)). However, thinning preserves disconnected components already present in the input and does not explicitly repair local breaks or eliminate every short remnant. Its output quality consequently depends on the continuity of the input mask, and the resulting skeleton is not necessarily ready for direct finite-element geometry construction.

The deep-learning methods predict wall centerlines from multiscale image features. The encoder-decoder architecture and skip connections of U-Net preserve fine positional detail (Fig. 5(e)), whereas the nested multiscale structure of U2-Net supports broad structural coverage (Fig. 5(f)). Nevertheless, neither architecture imposes a hard global constraint on skeleton connectivity or contour closure. Small local prediction gaps can therefore become disconnected components after skeleton extraction, even when the predicted pixels closely match the reference skeleton.

In contrast, TTGS combines wall-centerlines extraction with explicit node-cluster identification, path decomposition, and graph-based connectivity reconstruction (Fig. 5(g)). This procedure retains the wall-following advantage of direct skeleton extraction while imposing an explicit node-edge representation, thereby reducing positional errors relative to the Voronoi-based methods and suppressing short fragmented components through connectivity-aware reconstruction. Fig. 6 shows node-skeleton overlays at three locations: vascular bundles exhibit typical ring-like and radial connectivity patterns, whereas parenchyma regions mainly present polygonal mesh-like structures.

**Fig. 6:**
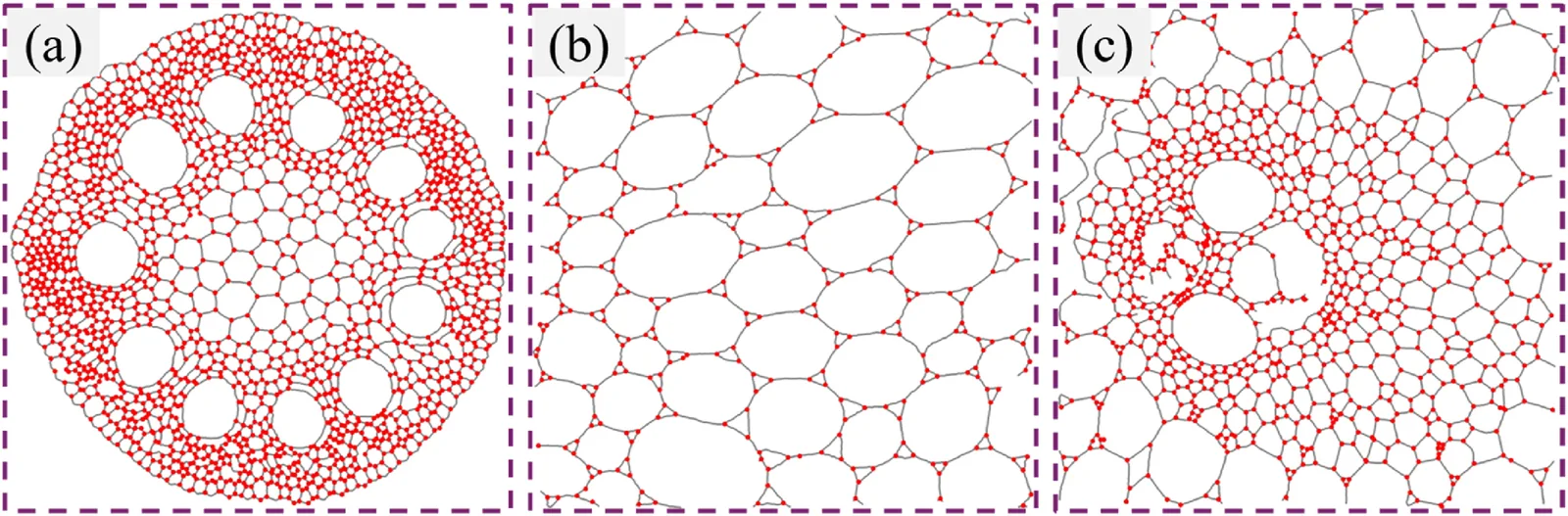
Node-skeleton overlays produced by TTGS at different locations (red dots: TTGS-identified nodes; gray lines: TTGS skeletons): (a) root tissues, (b) parenchyma, (c) vascular bundles.

### 3.4 Overall quantitative results, fragmentation screening, and topology-aware comparison

Different skeleton reconstruction methods exhibited distinct trade-offs between pixel-level agreement and network topology. To evaluate these differences, seven metrics were calculated for all seven methods across the 12 images: SC, GD_Mean, SER_Acc, BR, UIE, CC_F1, and DAJ_F1. Table 3 summarizes their overall performance across all tissue types.

**Table 3:** Overall skeleton reconstruction performance of the seven methods (mean ± SD, n = 12).

Method	SC (%)	GD_Mean (px)	SER_Acc (%)	BR (%)	UIE (−)	CC_F1 (%)	DAJ_F1 (%)
CVT	8.22 ± 6.33	49.82 ± 15.96	36.48 ± 20.87	0.00 ± 0.00	2.92 ± 5.18	35.22 ± 28.42	2.73 ± 2.95
FECVT	8.02 ± 5.98	33.42 ± 21.10	40.42 ± 23.93	0.00 ± 0.00	7.75 ± 4.11	36.35 ± 29.09	2.71 ± 2.90
ODWT	10.97 ± 7.52	21.79 ± 14.55	50.64 ± 26.53	0.00 ± 0.00	4.75 ± 2.67	49.04 ± 23.89	7.76 ± 7.49
Zhang-Suen	76.34 ± 10.55	2.12 ± 0.95	100.00 ± 0.00	63.62 ± 28.65	221.17 ± 132.85	85.47 ± 11.49	17.34 ± 12.39
U-Net	92.36 ± 4.28	0.68 ± 0.17	99.78 ± 0.28	57.59 ± 8.16	494.75 ± 315.19	57.72 ± 15.93	81.66 ± 8.52
U2-Net	90.33 ± 5.11	0.77 ± 0.16	99.69 ± 0.39	44.88 ± 15.15	833.00 ± 310.74	36.30 ± 22.49	80.39 ± 10.36
TTGS	44.04 ± 12.98	2.93 ± 1.04	94.49 ± 3.71	0.00 ± 0.00	5.00 ± 5.38	93.73 ± 5.56	92.75 ± 3.54

Although Zhang-Suen, U-Net, and U2-Net performed favorably in SC, SER_Acc, and GD_Mean, their BR values were 63.62%, 57.59%, and 44.88%, respectively. Their mean UIE values were also high, reaching 221.17, 494.75, and 833.00. These results show that favorable pixel-level performance does not necessarily correspond to a well-connected skeleton network. Outputs with BR > 0 require inspection and pruning of short fragments, together with reconnection where necessary, before entering the vectorization, meshing, and simulation workflow. CVT, FECVT, ODWT, and TTGS achieved BR = 0 under the predefined short-fragment criterion. Fig. 7 therefore compares the remaining six metrics for these four methods.

**Fig. 7:**
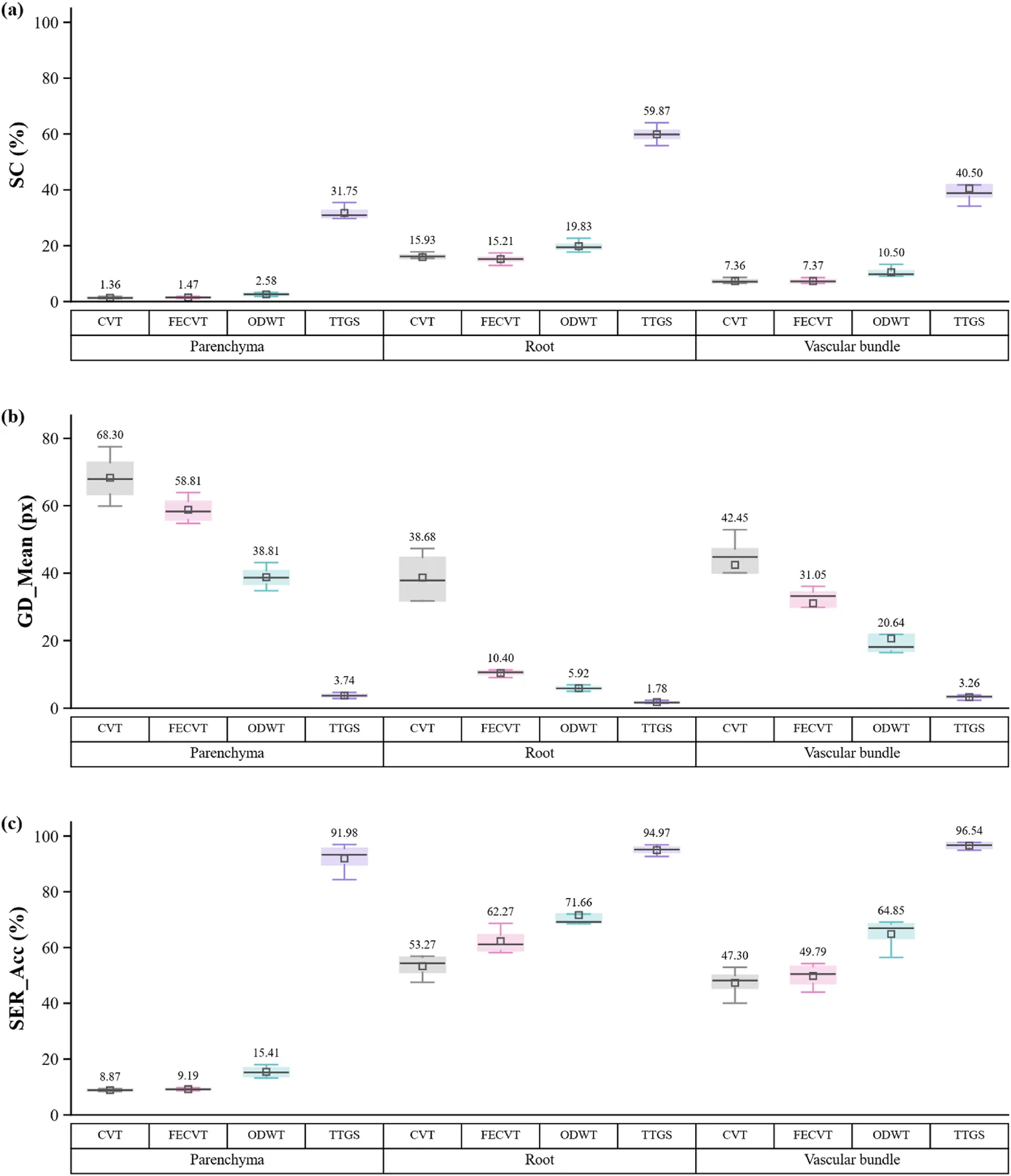

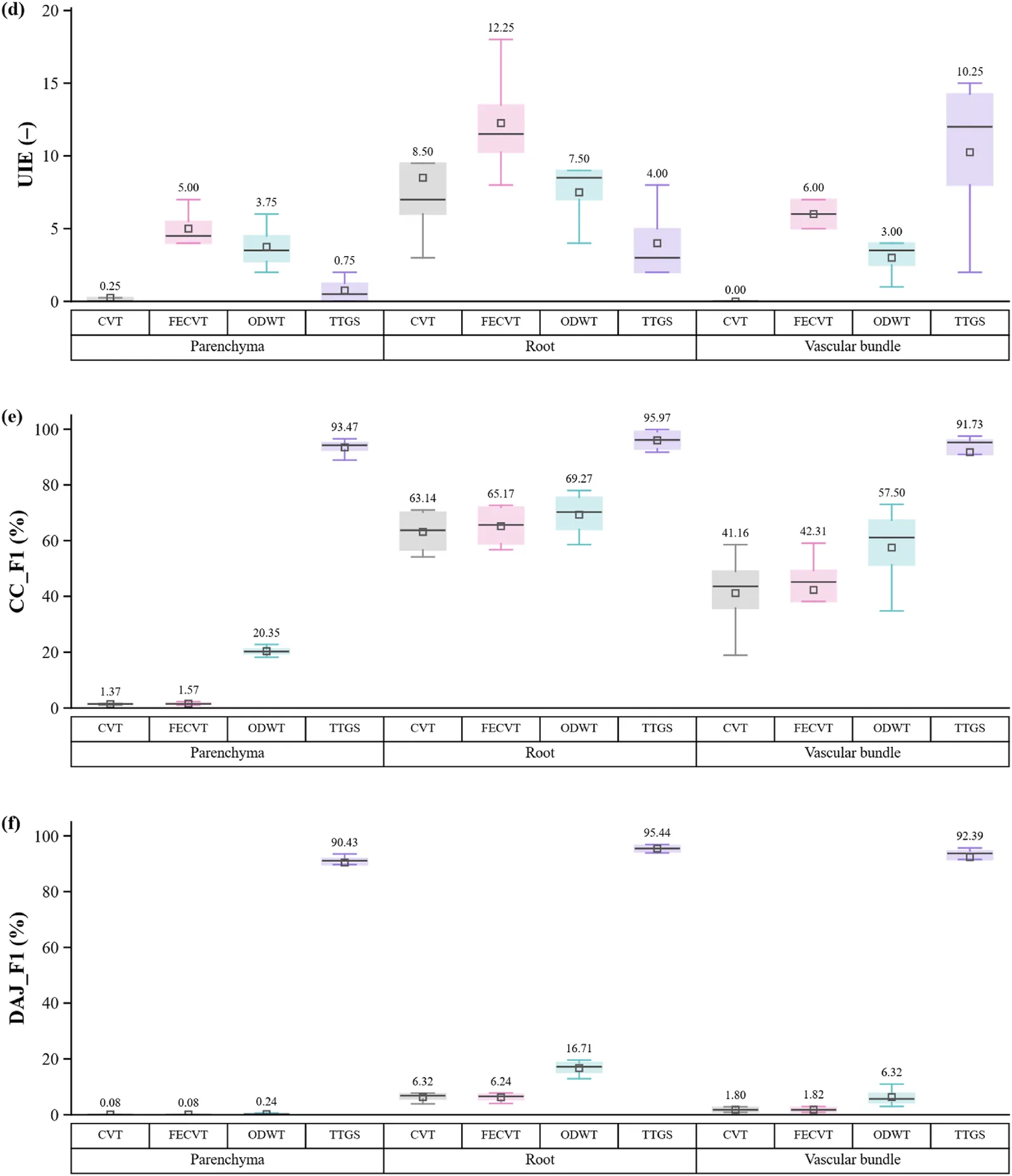
Evaluation metrics for the four methods satisfying BR = 0—CVT, FECVT, ODWT, and TTGS: (a) SC, (b) GD_Mean, (c) SER_Acc, (d) UIE, (e) CC_F1, and (f) DAJ_F1.

As shown in Fig. 7(a–c), TTGS simultaneously increased structural coverage, reduced geometric deviation, and retained a larger proportion of skeleton pixels within the cell-wall regions. Its SC values were 31.75%, 59.87%, and 40.50% for parenchyma, roots, and vascular bundles, respectively. The corresponding best values among CVT, FECVT, and ODWT were 2.58%, 19.83%, and 10.50%. TTGS also reduced GD_Mean to 3.74, 1.78, and 3.26 px, whereas the lowest corresponding values among the other three methods were 38.81, 5.92, and 20.64 px. Its SER_Acc reached 91.98%, 94.97%, and 96.54%, compared with the corresponding best values of 15.41%, 71.66%, and 64.85% for the other methods.

The spatial deviation maps in Fig. 8 further show that larger reconstruction errors were concentrated near tissue boundaries and transitions between large and small cells. These localized deviations were more pronounced for CVT, FECVT, and ODWT than for TTGS.

**Fig. 8:**
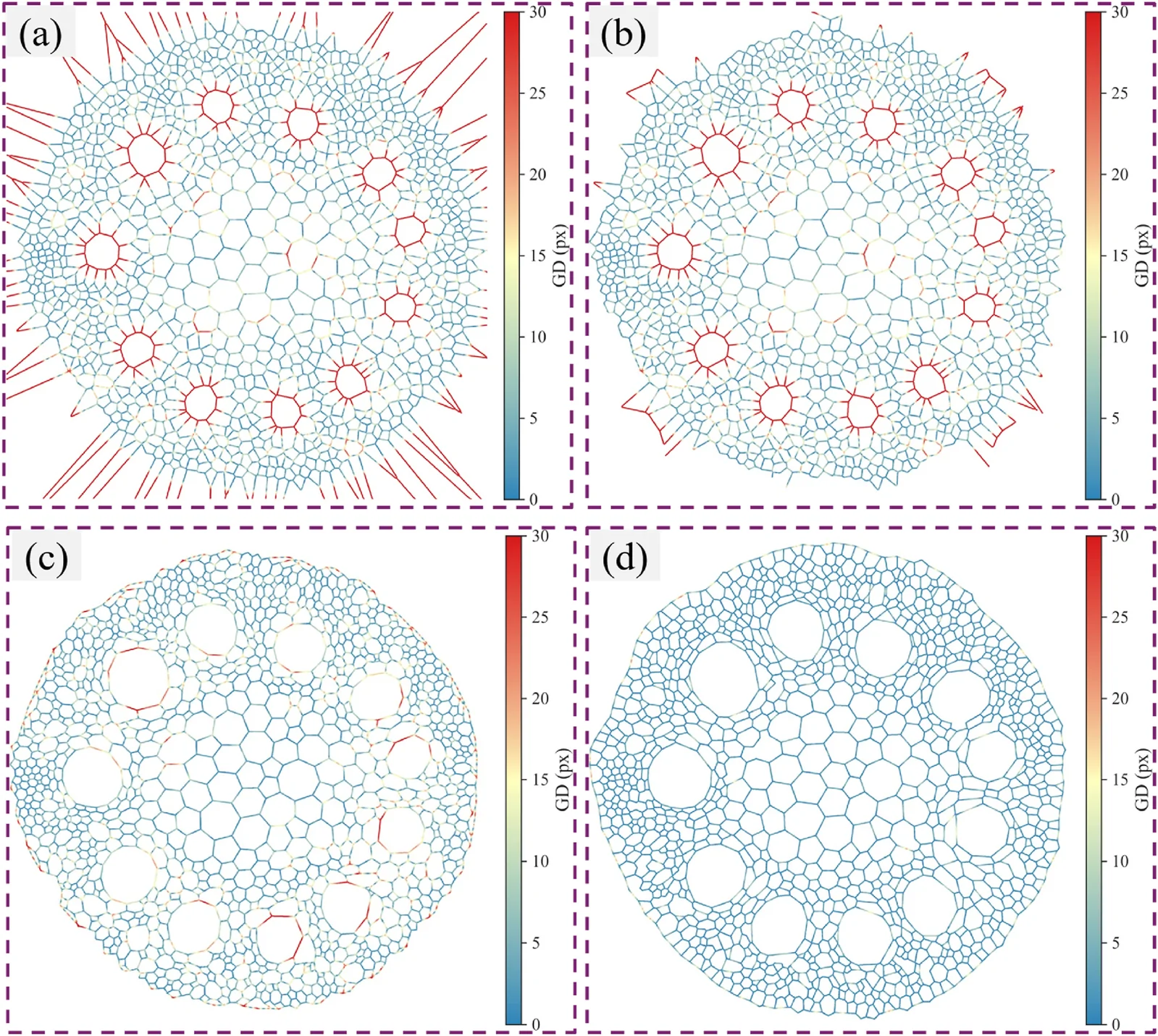
Visualization of skeleton GD_Mean for each method: (a) CVT, (b) FECVT, (c) ODWT, (d) TTGS.

TTGS did not consistently produce the lowest UIE across tissue types (Fig. 7(d)). Its UIE values were 0.75, 4.00, and 10.25 for parenchyma, roots, and vascular bundles, respectively. However, its advantage was more evident in the metrics describing network topology. TTGS achieved CC_F1 values of 93.47%, 95.97%, and 91.73% (Fig. 7(e)), whereas the best corresponding values among the other methods were 20.35%, 69.27%, and 57.50%. Its DAJ_F1 values reached 90.43%, 95.44%, and 92.39% (Fig. 7(f)), compared with 0.24%, 16.71%, and 6.32% for the best-performing alternatives. Thus, although some unmatched endpoints remained, TTGS preserved the principal closed contours and junction-degree structure more effectively.

Overall, among the four methods satisfying BR = 0, TTGS provided the most favorable balance between geometric fidelity and topological preservation. The combined SC, GD_Mean, and SER_Acc results showed improved structural coverage, centerline alignment, and wall-region consistency. The CC_F1 and DAJ_F1 results further demonstrated better preservation of closed contours and junction connectivity. These findings indicate that pixel-level agreement alone is insufficient for evaluating skeleton reconstruction. The TTGS skeletons provide a basis for subsequent vectorization and structural analysis, although geometric checking, mesh generation, and convergence assessment remain necessary before finite-element simulation.

To verify the applicability of the reconstructed network to finite-element analysis, a representative TTGS-generated vector network was directly imported into COMSOL Multiphysics for geometric reconstruction (Fig. 9(a)). The reconstructed geometry was extruded into a three-dimensional cell-scale model, in which different cell types and internal contact interfaces were assigned (Fig. 9(b)). Finite-element analysis was then performed under the prescribed loading and boundary conditions, producing a continuous stress distribution (Fig. 9(c)). These results validate the complete workflow from TTGS-based vector reconstruction to geometric modeling and finite-element solution.

**Fig. 9:**
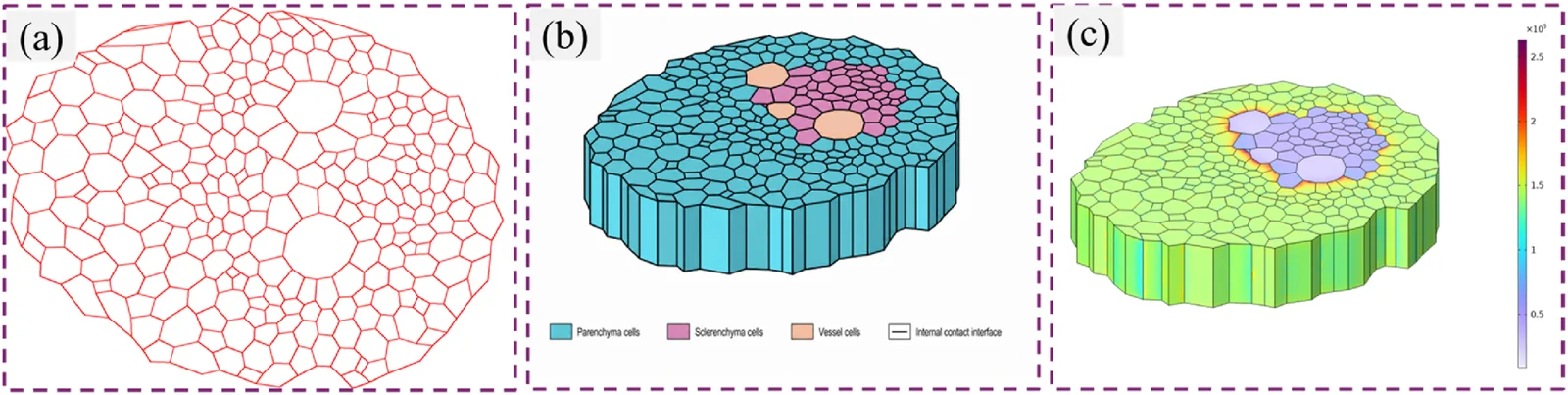
Finite-element application of the TTGS-reconstructed cell wall network: (a) vector network, (b) three-dimensional geometric model, and (c) stress distribution.

## 4 Conclusions

Accurate reconstruction of cell wall networks is a prerequisite for quantitative investigation of how microscale wall architecture is associated with tissue-level physiological functions, including mechanical support, hydraulic transport, and stress adaptation. To facilitate the conversion of plant-tissue micrographs into geometries useable for multiscale mechanical modeling, we propose a topology-preserving skeleton graph modeling method (TTGS) to address common issues in cell wall network reconstruction, including skeleton drift, cross-lumen spurious edges, and topological inconsistency. TTGS enables stable construction from a binary cell wall mask to a node-edge graph and engineering-oriented vector geometry, providing geometric and topological inputs for subsequent mesh generation and mechanical analysis.

TTGS follows a wall-to-skeleton strategy with explicit topological modeling. Node-cluster identification and merging, edge-segment decomposition, and path tracing are used to construct G = (V, E), preserving connectivity at complex junctions and enabling engineering-oriented DXF export. Consequently, among the tested methods satisfying BR = 0, TTGS provided the most favorable combination of geometric accuracy and network integrity for subsequent vectorization and finite-element modeling under the present experimental conditions, without requiring a separate short-fragment repair stage.

In comparative experiments on 12 images of sugarcane roots, parenchyma, and vascular bundles, TTGS achieved SC = 44.04 ± 12.98%, GD_Mean = 2.93 ± 1.04 px, SER_Acc = 94.49 ± 3.71%, and BR = 0%. TTGS further achieved UIE = 5.00 ± 5.38, CC_F1 = 93.73 ± 5.56%, and DAJ_F1 = 92.75 ± 3.54%, demonstrating favorable preservation of closed contours and junction-level connectivity. Among all tested methods with BR = 0, TTGS achieved the highest structural completeness and valid skeleton ratio and the lowest geometric deviation. Parameter sensitivity analyses further showed that TTGS remained relatively stable across the tested L_min_ and d_min_ ranges, with L_min_ = 30 px and d_min_ = 1 px retained for subsequent evaluation. A representative TTGS-generated vector network was successfully reconstructed as a three-dimensional finite-element model and solved to obtain a continuous stress distribution, demonstrating the feasibility of the workflow from skeleton reconstruction to finite-element analysis.

The present study has several limitations. Validation was limited to 12 microscopy images from three tissue regions of a single sugarcane cultivar under a controlled workflow. Broader tests across genotypes, species, staining protocols, imaging platforms, and image qualities are therefore needed to assess the general robustness of TTGS. In addition, straight-line parameterization may lose local curvature, and TTGS remained less complete than U-Net and U2-Net at the pixel level. Although independently verified, the thinning-initialized reference skeletons may still introduce some bias toward thinning-based methods. Future work will therefore focus on expanding the dataset, establishing multi-annotator reference skeletons, integrating deep-learning localization with TTGS-based topology repair, adopting spline-based parameterization, and extending the framework to 3D microscopy data.

## Data availability

The datasets, source code, model weights, testing instructions, and input and output files supporting this study are available at: https://figshare.com/s/8bb5f5e82384dd2ca9ee.

## Author contributions

Zaibin Wang and Zhenghe Luo drafted and revised the article. Tao Wu and Jiaoli Jiang contributed to study conceptualization and manuscript revision. Xueting Han, Qing Zhou, and Xiaojuan Lin collected data and performed data analysis. Yan Zhang prepared figures and tables. Tao Wu and Qingting Liu were responsible for supervision and funding acquisition. All authors read and approved the final article.

## Declaration of competing interest

The authors declare that they have no known competing financial interests or personal relationships that could have appeared to influence the work reported in this paper.

## Figures and Tables

**figs1:**
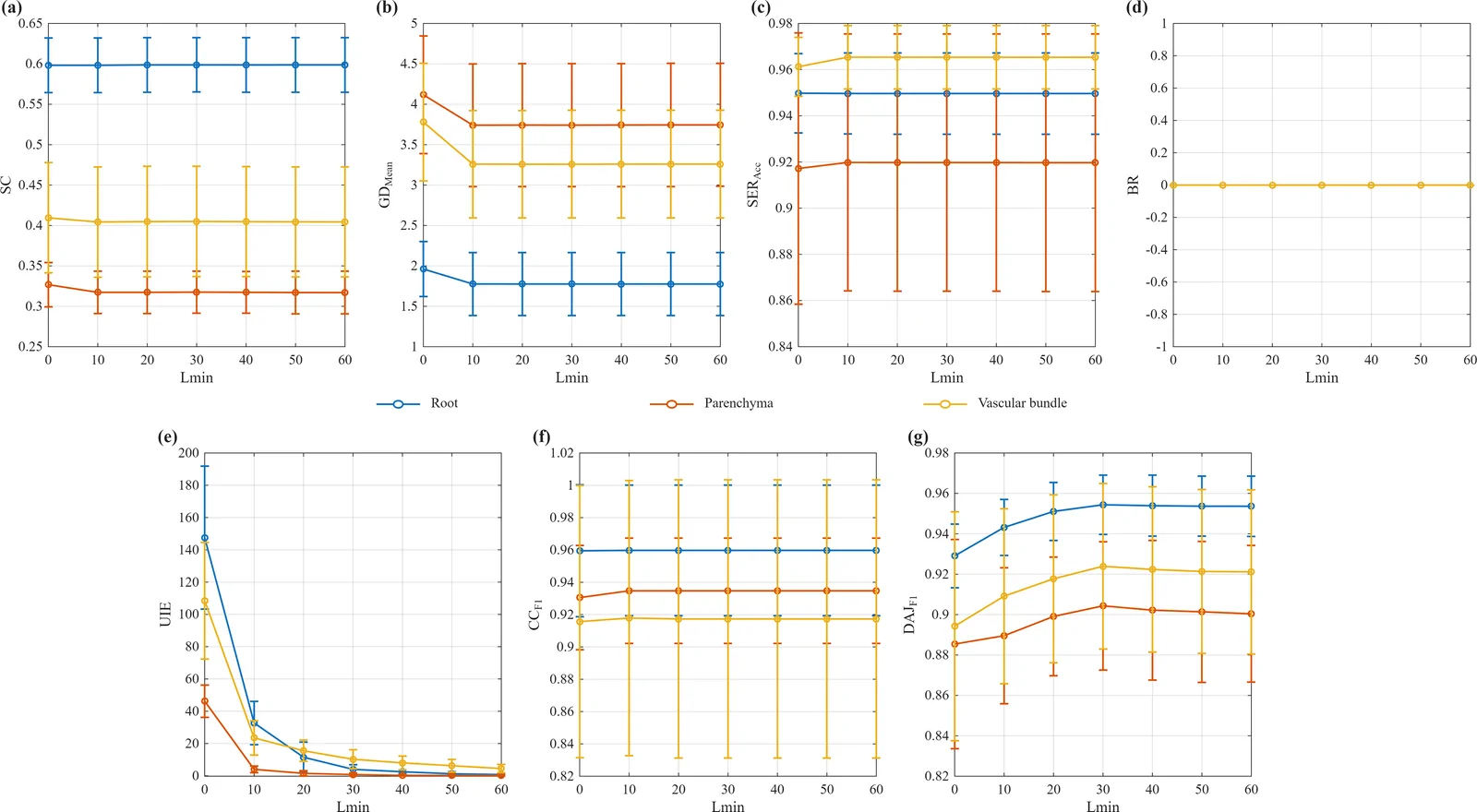
Effects of L_min_ on segmentation performance for root, parenchyma, and vascular bundle.

**figs2:**
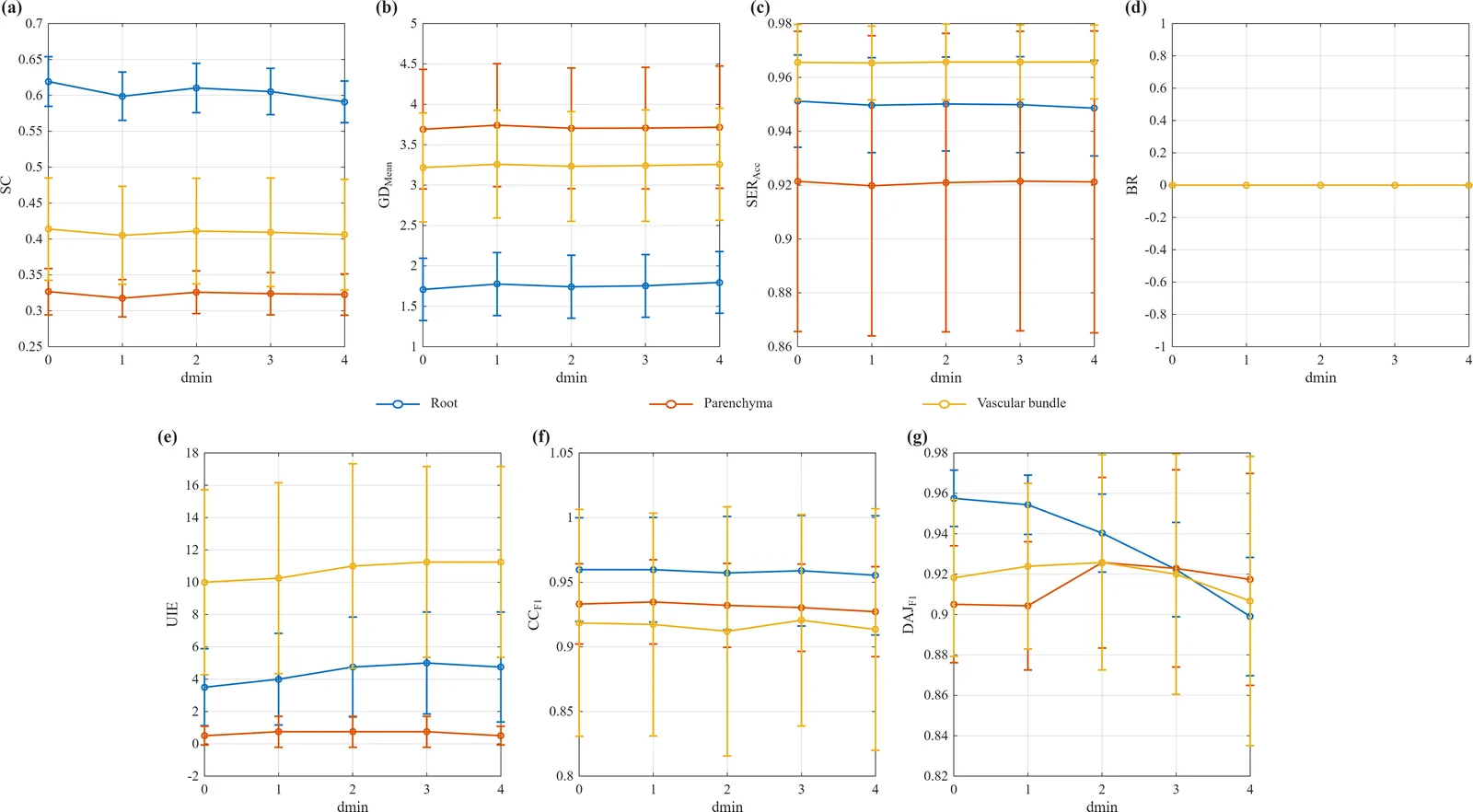
Effects of d_min_ on segmentation performance for root, parenchyma, and vascular bundle.
